# Effect of the Level of Coordinated Motor Abilities on Performance in Junior Judokas

**DOI:** 10.2478/v10078-011-0083-0

**Published:** 2011-12-25

**Authors:** Grzegorz Lech, Janusz Jaworski, Vladimir Lyakh, Robert Krawczyk

**Affiliations:** 1 The Department of Theory and Methodology of Combat Sports, the University School of Physical Education in Cracow; 2 The Chair of Anthropometrics, the University School of Physical Education in Cracow; 3 The Department of Sports Training, the Academy of Physical Education in Katowice

**Keywords:** combat sports, judo, coordination motor abilities

## Abstract

The main focus of this study was to identify coordinated motor abilities that affect fighting methods and performance in junior judokas. Subjects were selected for the study in consideration of their age, competition experience, body mass and prior sports level. Subjects’ competition history was taken into consideration when analysing the effectiveness of current fight actions, and individual sports level was determined with consideration to rank in the analysed competitions.

The study sought to determine the level of coordinated motor abilities of competitors. The scope of this analysis covered the following aspects: kinaesthetic differentiation, movement frequency, simple and selective reaction time (evoked by a visual or auditory stimulus), spatial orientation, visual-motor coordination, rhythmization, speed, accuracy and precision of movements and the ability to adapt movements and balance. A set of computer tests was employed for the analysis of all of the coordination abilities, while balance examinations were based on the Flamingo Balance Test. Finally, all relationships were determined based on the Spearman’s rank correlation coefficient. It was observed that the activity of the contestants during the fight correlated with the ability to differentiate movements and speed, accuracy and precision of movement, whereas the achievement level during competition was connected with reaction time.

## Introduction

Success in a judo fight is the result of a number of factors, only a few of which can be controlled in the training process. So far, much of the theory and practice of training has been emphasised a ‘champion model’ that focuses on the preferred body type, motor ability, and mental preparation as well as technical and tactical excellence. The use of this model has implications at the early stages of selection and qualification for sports careers; however, the following question remains: which key traits (strongly determined by genetic traits) should be emphasised? The world literature is dominated by publications that have examined levels of aerobic and anaerobic capacity (Little, 1991; Borkowski et al., 2001; Thomas et al., 2011), strength (Iwai et al., 2008) and body type (Franchini et al., 2007) in judo athletes at the highest levels. However, judo involves a great number of techniques and tactics that have yet to be fully studied. Hence, according to the authors of the present study, the processes of selection, planning and training should not be guided solely by champion model indexes. More attention should be directed to the development of coordination abilities along with technical and tactical development. This hypothesis was confirmed several times in analysis of judo contestants depending on body height (Lech et al., 2007) and aerobic and anaerobic capacity (Lech et al., 2010). In addition, in a study of basic coordination of motor skills in a group of senior athletes, favourable relationships were found between the level of technical and tactical excellence and the ability to differentiate movements. The correlation between performance during competition and speed, accuracy and precision of movements has also been demonstrated (Lech et al., 2007); however, it remains unknown which particular motor abilities affect the patterns of success in junior athletes.

A fundamental goal of this study was to evaluate the coordination abilities in junior judokas and to provide answers to the following research questions:

1. Does a correlation exist between performance during a fight and the level of chosen coordination abilities?
2. Is there a relationship between the effectiveness of actions and the level of chosen coordination abilities?
3. Is there a relationship between the sports level in the contestants and the level of chosen coordination abilities ?

## Material and Methods

The study covered 10 judokas in the following weight categories: under 73 kg (n=3), under 81 kg (n=2), under 90 kg (n=2) and under 100 kg (n=3). The subjects came from four separate clubs. Individual’s calendar age and competition experience was also taken into consideration for the groups.

Table 1 presents the age and training experience as well as the basic body type characteristics of the studied judokas.

During elimination in the lead up to the Junior Poland Cup (Bytom, 29 September 2007) and Junior Poland Cup (Warsaw, 20 October 2007), tournament matches fought by the contestants were recorded (n=58). Technical actions of individual contestants were evaluated during the analysis of the fights (analysis of selection matches encompassed only those with contestants who qualified for central competition). The competition was divided into two parts: phase I, represented by first two minutes of the fight, and phase II, represented by the third and fourth minutes. If extra time was used, it was included in the phase II of the analysis. All technical actions evaluated by judges and referees as well as ineffective actions, which were not given points by the judges, were considered for the study. When a contestant threw his opponent out of balance (‘flying phase’ was observed), the actions were assigned a 0 note. In total, 137 technical actions were recorded. Based on the collected data, indexes that determine the activity and effectiveness of actions among the study participants were calculated. The activity index (WA) was calculated from the following formula: WA=ΣA/NW, where ΣA is a total of the attacks and NW is the number of fights the contestant fought. The activity index calculated for phase 1 was WA1, with WA2 for phase 2. Another index, RWA (difference in the activity index), was also calculated to reflect the variability of activity during competition. It was calculated as follows: RWA=WA1–WA2. The effectiveness index (WS) is an arithmetic mean of the notes for attacks (WS1 as calculated for phase 1 of the match and WS2 for the second phase). The difference in the effectiveness index was calculated from the following formula: RWS=WS1–WS2.

Sports performance was evaluated as a total number of points scored by the contestants during both tournaments according to the following point scale:

- preliminary fights: 1st: 1st place – 3 points, 2nd place – 2 points, 3rd place – 1 point, 5th place – 0.5 points; and
- central competition: 1st place – 7 points, 2nd – 5 points, 3rd – 3.5 points, 5th – 1.5 points, and 7th – 0.5 points.

Another aspect of the work was to determine the level of motor ability coordination. The examination was performed equidistant from the first competition and the second tournament.

Based on the recent classifications of coordinated motor abilities, the present study covered the following parameters: kinaesthetic differentiation of movements, movement frequency, simple reaction time (to visual and auditory stimuli), selective reaction time, spatial orientation, visual-motor coordination, rhythm, speed, accuracy and precision of movements and ability to adapt movements. Examinations were carried out in a room that was quiet and relaxing for all the subjects included in the study. A set of computer tests using the Toshiba Satellite R15 touch screen was employed for the analysis of the coordination abilities. The computer tests were characterised by a sufficient level of reliability (ICC ranged in general from 0.60 to 0.93) for each type of the above measurement tools. The Flamingo Balance Test was also employed for evaluation of balance according to the European test battery Eurofit (1993), and the STATISTICA PL v. 6.0 software package was employed for the analysis of the results. Spearman’s rank correlation coefficient and Mann-Whitney U-test were also used during the study.

## Results

Table 2 presents the values of coefficients that determine the fight.

The analysis of the activity index (WA) revealed that contestants performed from 1.0 to 3.5 technical actions per fight, but a comparison of the activity within the individual periods of competition revealed a considerable difference. The studied group included both judokas whose activity increased in the second part of fight (minimum value of RWA =−1.7) and those who performed fewer actions (maximum value of RWA=0.5). The mean RWA (−0.5) suggests a tendency for increased activity in the second part of fight.

The mean value of the effectiveness index (WS) in the studied group amounted to 3.4. Similarly to the activity index, individual judokas varied considerably (minimum = 2.4 points, maximum = 6.8 points). The analysis of the RWS value (0.8 points) revealed a tendency towards a decline in the mean value of the points given in the second part of the fight. However, in individual cases, contestants demonstrated a considerable rise in effectiveness (−3.2) in the 3rd and 4th minutes of match.

Although differentiation occurred, on average, the level of achievement (PO) was 3.3 with the lowest participant at 1 point and the highest participant at 6 points. Individual cases reveal that the biggest differentiation amongst the judokas was observed in movement (test No. 17, V=75.9), spatial orientation (test No. 25, V=73.4) and visual-motor coordination, (test No. 23, V=69.3).

Reaction time varied the least among the group as follows: minimum reaction time to visual stimulus (test No. 3, V=6.7), mean reaction time to visual stimulus, minimum reaction time to auditory stimulus (tests No. 4 and 6, V=8.7) and also minimum reaction time and mean complex reaction time (tests No. 9 and 10, V=9.6).

Table 4 compares statistically significant values of Spearman’s rank correlation coefficients calculated between the results of coordination tests and the sports performance in the studied group of contestants.

Analysis of the value of Spearman’s R coefficient for WA revealed that its value was negatively correlated to the ability to differentiate movements (high correlation, Spearman’s coefficient: R=−0,7).

While the examination of WA1 (activity index for the first part of match) revealed a positive correlation to mean reaction time (Spearman’s R coefficient=0.65) and maximum reaction time (Spearman’s R coefficient=0.64), overall, a negative correlation was observed for WA1 and the time to complete labyrinth to the left test (Spearman’s R coefficient=−0.7).

During the second part of a fight (WA2), a high negative correlation was observed with regards to the frequency of hand movement (Spearman’s R coefficient=−0.67). Additionally, a high positive correlation in the number of mistakes in the labyrinth to the right test was also observed (Spearman’s R coefficient=0.63).

A very high positive correlation was found between the value of RWA (difference in the activity index) and the frequency of hand movements (Spearman’s R coefficient=0.82).

Effectiveness in the first part of a fight positively correlated with the result of the kinaesthetic differentiation test (Spearman’s R coefficient=0.82; very high correlation). While the effectiveness in the second part of a fight (WS2) was significantly correlated with the number of mistakes observed during the optional test (SO) (very high positive correlation, Spearman’s R coefficient=0.75). A negative correlation was observed between the level of sports performance (PO) and the mean complex reaction time (Spearman’s R coefficient=−0.798) and the maximum complex reaction time (Spearman’s R coefficient=−0.69). Overall, the level of achievement correlated to the difference in mistakes found during performance of labyrinth to the right and labyrinth to the left (Spearman’s R coefficient=0.67).

## Discussion

The ICC reliability coefficients in the authors’ test battery determined based on testretest results ranged from 0.60 to 0.93. The proposed computer tests were characterised by the appropriate reliability for this type of measurement tools (Kirkendall et al., 1987; Domholdt, 2000). Comparison of the obtained results of reliability coefficients with the available data reveals that the results are similar to the data from the Vienna Test System (http://www.schuhfried.at/wiener-testsystem-wts/2011). The authors of the system demonstrated that coefficients of test reliability range from 0.50 to 0.98. For example, the reliability in the tests of shape learning, number learning and tapping was from 0.50 to 0.55, whereas for reaction time, motor time in reaction analysis and sustained attention, the reliability amounted to as much as 0.99. In addition, in the study by Juras et al. (2008), ICC coefficients for the test of maximal sway (functional balance) amounted to 0.85.

Coordinated motor abilities have long been the focus for coaches and players of team sport games. Literature on this subject is broadly available with a dedicated webpage (www.koordinationstraining.com 2011) that offers materials, workshops and training programs for coaches and physical education teachers. The site provides well-tested concepts of specific coordination training schemes for football, basketball, volleyball and handball. Remarkably, few studies have examined the importance of coordination in combat sports. Interesting investigations of the effect of practising judo onmotor coordination were carried out by May et al. (2001), and the results of a multivariate analysis demonstrated that after 6 and 12 months of training in a group, judokas showed a statistically significant improvement in balance, whereas no significant increase was observed in tapping range and time of reaction to visual or auditory stimulus (determined based on the Vienna Test). The presented results are in contrast to those observed by Drida et al. (2009). After a 2- year judo training programme, boys aged 11 to 15 years from the Serbian province of Vojvodina exhibited better body builds and better results for coordination abilities compared to untrained peers.

The unfavourable correlations found in the present study are likely to be caused by the specific nature of training in this sport. General training (predominantly using exercises that emphasise strength and endurance development) seem to considerably limit development of motor coordination, whereas specific exercises (technique training, participation in competitions, sparring) lead to the development of its particular components.

A comparison of tests results obtained among the studied subjects and the tests for seniors (Lech et al., 2007) revealed a statistically significant difference observed only in case of minimum complex reaction time (test No. 9); juniors obtained better results in this test.

A favourable correlation was also observed between technical and tactical level indexes and the ability to differentiate movements (WA – kinaesthetic differentiation – anticipation), as well as speed, accuracy and precision of movements (WA1 – time for completion of labyrinth to the left test).

It was also observed that sports success correlated to reaction time (mean time of complex reaction and maximum time of complex reaction).

Comparison of the correlations to the results for seniors revealed that the factors significant to the course of fight and the level of sports performance are as follows:

- The ability to differentiate movements (in the group of seniors, it correlated with the effectiveness in the first part of match);
- speed, accuracy and precision of movements (number of mistakes in this test was correlated with the level of achievement); and
- reaction time (in the group of seniors, the mean time of complex reaction correlated with the effectiveness in the first part of match).

Summarising the comparison above, one can conclude that a higher number of elementary coordinated motor abilities impacts the technical and tactical level of contestants.

The reasons for the differences lie in the specific nature of training, which has a decisive effect on the way the judokas fight. The authors’ coaching practice demonstrated that the group of juniors focus more on technique improvement (in most of cases, the contestants have not gained a comprehensive technical preparation yet), whereas older contestants seem to prefer exercises focused on technical and tactical development (with particular focus on task-oriented sparring).

No relationships were found between the level and indexes of fight course and sports level of the contestants. The correlation between the results of Flamingo Balance and the effectiveness of the contestants’ actions was at the threshold of statistical significance (p=0.083, r=−0.57), indicating tendencies towards the improvement in the effectiveness of activities with an increase in balance abilities. However, the adopted level of significance (p<0.05) does not allow to evaluate this relationship in the results. Lack of relationships between balance abilities and rank in judo contestants was also demonstrated by Hrysomallis (2011). Authors of the present study agree with the Hrysomallis’ view that ‘balance training can be a significant component to the usual training of non-elite athletes to enhance certain motor abilities’.

## Conclusions

Favourable and statistically significant relationships were obtained between the following aspects:

1. Activity level of the contestants and
  - the ability to differentiate movements
  - speed, accuracy and precision of movements.
2. Sports level and complex reaction time.

## Figures and Tables

**Table 1:** Age, experience and basic parameters of somatic build in study participants (n=10)

Variables	*x̄*	Min.	Max.	SD
Age (years)	17.5	16.0	18.0	0.71
Experience (years)	8.4	7.0	11.0	1.17
Body height (cm)	180.4	173.0	186.0	3.63
Body mass (kg)	85.8	71.9	101.2	10.50
Fat free mass (kg)	72.1	64.4	79.0	5.51

**Table 2:** Characteristics of the indexes that determine activity, effectiveness and the rank of study participants (n=10)

	*x̄*	Min.	Max	SD
WA	2.1	1.0	3.5	0.63
WA1	1.0	0.3	2.0	0.45
WA2	1.6	0.7	2.2	0.56
RWA	−0.5	−1.7	0.5	0.73
WS	3.4	2.4	6.8	1.37
WS1	4.0	2.2	7.0	2.02
WS2	3.2	0.8	6.8	2.06
RWS	0.8	−3.2	6.3	2.95
PO	3.3	1.0	6.0	1.51

**Table 3:** Parameters for the determination of coordination motor abilities (n=10)

CMA	Test No.	Parameter / units	*x̄*	Min.	Max.	V
Ability to differentiate movements	1	Kinaesthetic differentiation – anticipation / pixel	36.8	16	64	37.8

Frequency of movements	2	Hand movements frequency (tapping) / n	43.2	35	57	17.8

Reaction time	3	Minimum reaction time (visual stimulus) / ms	233	220	260	6.7
	4	Mean reaction time (visual stimulus) / ms	247	225	285	8.7
	5	Maximum reaction time (visual stimulus) / ms	270	240	340	13.2
	6	Minimum reaction time (auditory stimulus) / ms	196	180	240	8.7
	7	Mean reaction time (auditory stimulus) / ms	212	188	255	10.9
	8	Maximum reaction time (auditory stimulus) / ms	234	200	300	14
	9	Minimum complex reaction time / ms	250	220	300	9.6
	10	Mean complex reaction time / ms	362	316	432	9.9
	11	Maximum complex reaction time / ms	521	410	620	14

Rhythmization ability	12	Movement rhythmization / ms	93	58	240	58.9

Speed. accuracy and precision of movements	13	Labyrinth to the left / s	46	37	57	16.2
	14	Labyrinth to the right / s	40.8	31	57	21.2
	15	Labyrinth to the left / mistakes n	11.8	3	24	55.8
	16	Labyrinth to the right / mistakes n	8.8	1	17	57.6

Motor adjustment	17	Difference between the direction to the right and to the left / s	5.6	0	13	75.9
	18	Difference between the direction to the right and to the left / mistakes n	6.2	1	13	58.3

Visual-motor coordination	19	Optional / s	74.4	59	95	13.7
	20	Forced / errors n	69	31	94	31.9
	21	Forced / correct n	31	6	69	71
	22	Eye-hand coordination / s	69.1	46	94	21
	23	Eye-hand coordination / mistakes n	19	4	42	69.3

Spatial orientation	24	Optional / s	54.3	43	64	10.8
	25	Optional /errors n	1.6	0	3	73.4

Reaction to moving objects	26	Forced / correct n	32,9	14	44	28.4
	27	Forced / errors n	17.1	6	36	54.6

Balance	28	Number of attempts to stand on the beam / n	6.8	2	12	50.8

**Table 4:** Statistically significant (p<0.05) values of rank correlation coefficient calculated between the results of coordination tests and sports performance in the studied group of contestants. (n=10)

Correlated coefficients		Spearman’s rank Coefficient	(N-2)	Level of significance
WA &	Kinaesthetic differentiation – anticipation (pixels)	−0.67	−2.54	0.034
WA1 &	Mean reaction time to auditory stimulus (ms)	0.65	2.43	0.041
WA1 &	Maximum reaction time to auditory stimulus (ms)	0.64	2.33	0.048
WA1 &	Labyrinth to the left (s)	−0.72	−2.93	0.019
WA2 &	Frequency of hand movements (n)	−0.67	−2.57	0.033
WA2 &	Labyrinth to the right (n mistakes)	0.66	2.50	0.037
RWA &	Frequency of hand movements (n)	0.82	3.99	0.004
WS1 &	Kinaesthetic differentiation (pixels)	0.73	3.02	0.017
WS2 &	/SO/ Optional (n mistakes)	0.75	3.23	0.012
PO &	Mean complex reaction time (ms)	−0.80	−3.76	0.006
PO &	Maximum complex reaction time (ms)	−0.69	−2.66	0.029
PO &	Difference between the direction to the right and to the left / adaptation (n mistakes)	0.67	2.53	0.035

Favourable correlations are written in bold (better results in coordination motor abilities were associated with a favourable value of the index determining sports performance).
